# Lung cancer associated with cystic airspaces: an underrecognized condition

**DOI:** 10.36416/1806-3756/e20250385

**Published:** 2026-03-05

**Authors:** Edson Marchiori, Bruno Hochhegger, Gláucia Zanetti

**Affiliations:** 1. Universidade Federal do Rio de Janeiro, Rio de Janeiro (RJ) Brasil.; 2. University of Florida, Gainesville (FL) USA.

A 31-year-old nonsmoking male athlete underwent an annual follow-up chest X-ray examination, which showed a cavitary lesion in the right lung. The patient was asymptomatic, with no respiratory complaints, and laboratory tests for tuberculosis and fungi were negative. A chest CT scan showed a cystic lesion with irregularly thick walls (Figure 1A), suggestive of an inflammatory/infectious process given the young age and smoking status of the patient. Bronchoscopy with bronchoalveolar lavage was negative. A follow-up CT scan performed 4 months later showed an increased solid component in the cavity wall (Figure 1B). A PET/CT scan showed significant fluorodeoxyglucose uptake (Figure 1C). Lesion biopsy led to a diagnosis of adenocarcinoma. 

**Figure 1:**
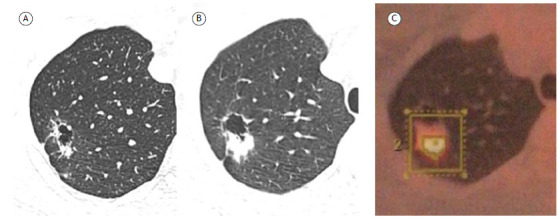
In A, axial unenhanced chest CT image showing a cystic lesion with irregularly thick walls in the right upper lobe of the lung and a small nodular component in the posterior region. In B, follow-up CT image obtained 4 months later, showing a significant increase in nodule size. In C, PET/CT image showing significant fluorodeoxyglucose uptake, with a standardized uptake value of 12.2.

Lung cancer associated with cystic airspaces (LCCA) is characterized by cystic cavities in or around the solid component seen on imaging (cyst wall thickening or nodularity). Because LCCA may progress indolently, long-term follow-up is often indicated. Indeterminate lesion growth or development of a solid or ground-glass component over time is more predictive of potential malignancy than are changes in cyst size.^1^ ^-^ ^3^ In thoracic oncology, LCCA is a relatively uncommon but important entity that is underdiagnosed on imaging. Familiarity with the imaging features and temporal evolution of LCCA can minimize delays in lung cancer diagnosis. 

## References

[1] Valsecchi C, Petrella F, Freguia S, Frattini M, Argentieri G, Puligheddu C (2025). Lung Cancers Associated with Cystic Airspaces. Cancers (Basel).

[2] Mendoza DP, Heeger A, Mino-Kenudson M, Lanuti M, Shepard JO, Sequist LV (2021). Clinicopathologic and Longitudinal Imaging Features of Lung Cancer Associated With Cystic Airspaces A Systematic Review and Meta-Analysis. AJR Am J Roentgenol.

[3] Penha D, Pinto E, Taborda-Barata L, Irion K, Marchiori E (2020). Lung cancer associated with cystic airspaces a new radiological presentation of lung cancer. J Bras Pneumol.

